# Mate choice for a male carotenoid-based ornament is linked to female dietary carotenoid intake and accumulation

**DOI:** 10.1186/1471-2148-12-3

**Published:** 2012-01-10

**Authors:** Matthew B Toomey, Kevin J McGraw

**Affiliations:** 1 School of Life Sciences, Arizona State University, Tempe, AZ 85287-4501 USA; 2 Division of Neuropathology, Washington University School of Medicine, MO 63110, Saint Louis

## Abstract

**Background:**

The coevolution of male traits and female mate preferences has led to the elaboration and diversification of sexually selected traits; however the mechanisms that mediate trait-preference coevolution are largely unknown. Carotenoid acquisition and accumulation are key determinants of the expression of male sexually selected carotenoid-based coloration and a primary mechanism maintaining the honest information content of these signals. Carotenoids also influence female health and reproduction in ways that may alter the costs and benefits of mate choice behaviours and thus provide a potential biochemical link between the expression of male traits and female preferences. To test this hypothesis, we manipulated the dietary carotenoid levels of captive female house finches (*Carpodacus mexicanus*) and assessed their mate choice behavior in response to color-manipulated male finches.

**Results:**

Females preferred to associate with red males, but carotenoid supplementation did not influence the direction or strength of this preference. Females receiving a low-carotenoid diet were less responsive to males in general, and discrimination among the colorful males was positively linked to female plasma carotenoid levels at the beginning of the study when the diet of all birds was carotenoid-limited.

**Conclusions:**

Although female preference for red males was not influenced by carotenoid intake, changes in mating responsiveness and discrimination linked to female carotenoid status may alter how this preference is translated into choice. The reddest males, with the most carotenoid rich plumage, tend to pair early in the breeding season. If carotenoid-related variations in female choice behaviour shift the timing of pairing, then they have the potential to promote assortative mating by carotenoid status and drive the evolution of carotenoid-based male plumage coloration.

## Background

Female mate preferences have led to the emergence of extremely elaborate and diverse male ornamentation in many animals (reviewed in [1]). A robust framework exists for understanding how traits and preferences coevolve at the population genetic level [2-5]. These models predict that sexual selection and the assortative mating of attractive males and choosy females inevitably leads to a positive genetic covariance between male trait and female preference. Yet, the physiological pathways that translate such genetic information to mating behaviors are largely unknown. These physiological mechanisms have the potential to profoundly shape the rate and direction of coevolution if they have mutually pleiotropic effects on the expression of a trait and the preference for that trait.

Sexually selected carotenoid-based male coloration appears in a diversity of taxa, from crabs (*Callinectes sapidus*; [6]) and fish (*Poecilia reticulata*; [7]) to birds (*Carpodacus mexicanus*; [8]), and has become a model system for understanding the costs, benefits, and evolution of male sexual trait expression [9]. Carotenoids are a class of pigment molecules that vertebrates are unable to produce endogenously and must acquire directly or indirectly from plants, bacteria, or fungi [10]. Carotenoids can promote immune function [11] and alleviate oxidative stress [12]; but see [13]. Thus, carotenoid-based coloration reveals information about male quality (i.e. diet, health) and female preferences for intense carotenoid-based coloration have been demonstrated in a number of taxa (especially birds; reviewed in [14]).

Although the costs and benefits of carotenoid pigments for male coloration have dominated the attention of behavioral ecologists, carotenoids have the potential to shape female choice behavior, through their direct influence on female physiology. Similar to males, carotenoids can enhance immune system responsiveness and antioxidant protection in females (e.g. [12,15]) which may permit investment in costly mate choice behaviors [16]. Carotenoids are also particularly important to breeding female birds because they are an essential component in egg yolks [17]. Carotenoid supplementation of females enhances egg production, yolk carotenoid accumulation, and subsequent embryo development and offspring quality [18-22]. Thus, the reproductive condition of female birds may be linked to the environmental and physiological availability of carotenoids. We hypothesize that traits underlying carotenoid acquisition and accumulation provide a mechanistic link between female choice behavior and male coloration.

Carotenoids may also influence mate choice for colorful traits through their direct influence on the visual systems of birds. In the avian retina, carotenoid pigments accumulate within oil droplets located between the inner and outer segments of the single cone photoreceptors [23,24]. In this position, carotenoids modify the spectral sensitivity of the cone in a way that is predicted to enhance color discrimination and color constancy [25]. Similar to plumage coloration, carotenoid accumulation in the avian retina is constrained by diet and health [26-31], and variation in retinal accumulation is linked to aspects of visual discrimination [32]. Therefore, carotenoid accumulation in the retina may influence a female's ability to discriminate the coloration of potential mates.

In an indirect manner, ecological changes in the availability of dietary carotenoids have the potential to change the value of carotenoid-based colors as indicators of male quality and the benefits to females for choosing those traits [33,34]. Specifically, carotenoid-based male signals in a carotenoid-rich environment may not be useful indicators of quality because it is relatively easy for males to acquire all of the carotenoids they need to become colorful. Consistent with this hypothesis, Grether et al. [34] observed that female guppies reared on a carotenoid-limited diet had significantly stronger preferences for male carotenoid-based coloration then females reared on a carotenoid-rich diet. This result suggests that dietary carotenoid levels provide females with information about environmental carotenoid availability that they may use to weigh the value of carotenoid-based male signals. However, physiological mechanisms behind this environmentally tuned response are unknown.

To investigate influence of carotenoids on female mate choice, we manipulated dietary carotenoid intake, quantified physiological accumulation of carotenoids, and examined mate selection behaviors of female house finches (*Carpodacus mexicanus*). The house finch is a model species for the study of sexually selected carotenoid-based coloration; males have plumage that varies from drab yellow to brilliant red depending upon dietary carotenoid access, health, and genetic quality [35]. Thus, male coloration is considered an honest indicator of male quality and, in nearly all populations, females prefer brilliant red males [36,37] but see [38]. Although these population-level preferences for male coloration are clear, individuals within a population may vary in their responsiveness, discrimination, and the strength of their preferences, in ways that may alter the intensity and direction of sexual selection [16,39,40]. Because mate choice is a complex behavior with components that may be differentially influenced by physiological or environmental conditions, we examined three specific components of choice.

Mate choice behavior can be initially divided into two components: 1) the preference function and 2) choosiness [39-41]. The preference function is the slope of the relationship between a female's response and the level of expression of the male trait [39-41]; steeper slopes indicate stronger preferences. Choosiness reflects the effort invested into mate choice by the female and can be further divided into two components: 1) responsiveness and 2) discrimination [41]. Responsiveness is the mean level of response by a female to all males (i.e. general mating interest), and discrimination is the variance in the female's response among the males she has sampled [41]. A high level of discrimination indicates that a female is biasing her response toward a specific male, while a low level of discrimination indicates a similar response to all males.

To examine the influence of carotenoids on these three components of mate choice, we captured female finches prior to the breeding season, maintained them in captivity, and fed them high- or low-carotenoid diets. We then presented these females with males that were manipulated to vary from yellow to red and measured the association time of the females with the males. Association time has been shown to be a reliable indicator of female choice in house finches [42]. From these observations, we calculated the preference function, responsiveness, and discrimination for each female [41,43]. We measured plasma carotenoid levels before, during, and after carotenoid supplementation and retinal carotenoid levels at the conclusion of the study, using high performance liquid chromatography [44]. Because all females were maintained on a very low carotenoid diet for two months before carotenoid supplementation, we considered our initial measure of plasma carotenoid levels to be an indicator of a female's ability to accumulate carotenoids from a limited diet [45]. Therefore, if female preference for carotenoid-based coloration is linked to carotenoid accumulation and availability, we predicted that both the preference function and choosiness would be positively correlated with pre-supplementation plasma carotenoid levels and significantly increased among carotenoid-supplemented females. If retinal carotenoids affect a female's ability to discriminate among potential mates, we predicted that retinal carotenoid levels would be positively correlated with the level of mate discrimination and with the repeatability of the preference function between successive choice trials. Alternatively, the environmentally contingent carotenoid indicator model of Grether et al. [34] predicts that strength of female color preference should be negatively related to carotenoid availability and accumulation.

## Results

### Female carotenoid accumulation, body condition, and activity rate during mate choice

**Figure 1:**
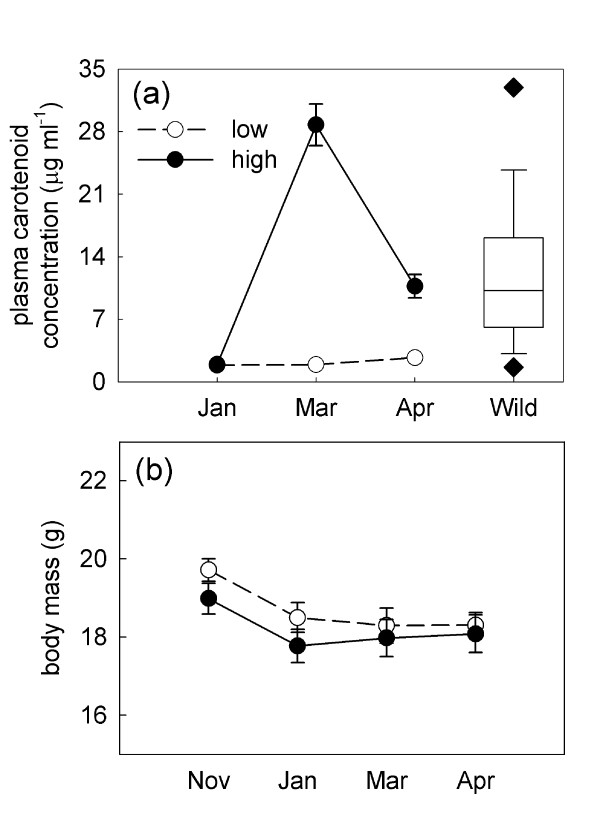
**Female plasma carotenoid levels and body mass throughout the diet manipulation**. (a) Mean ± s.e. plasma carotenoid concentrations of the high- and low-carotenoid diet female house finches throughout the study. Carotenoid supplementation began after the first sample was obtained (Jan.), and the mate choice tests were carried out in the period between the Mar. and Apr. samples. The box plot represents that carotenoid levels of wild female plasma measured in Jan.-Apr. 2005 and 2006 ([44], McGraw unpublished data) and is given for comparison. (b) Body mass of the high- and low-carotenoid finches at capture (Nov.) and throughout the study. Open symbols represent the low-carotenoid females and closed symbols represent the high-carotenoid females.

In January, prior to carotenoid supplementation, the plasma carotenoid levels of the female finches did not differ significantly between the treatment groups (Tukey's post hoc *p *= 1.00, Figure 1a). The high-carotenoid diet significantly increased plasma carotenoid levels compared to initial levels and females fed the low-carotenoid diet (time × diet: *F*_2,50 _= 108.12, *p *< 0.0001, Figure 1a). Before and during supplementation, the plasma carotenoid levels of the females were within the range of variation observed amongst wild birds at the same time of year (mean: 13.2 ± 1.1 μg ml^-1^, range: 0.023-93.2 μg ml^-1^, n = 157, Figure 1a,[44], McGraw unpublished data). Following the eight-week carotenoid supplementation period, we returned all of birds to the low-carotenoid diet in an effort to minimize differences in circulating carotenoid levels during the mate choice trials. Our goal was to isolate the persistent diet-induced changes in retinal carotenoid accumulation from the more labile plasma carotenoid levels [30] and to test the specific role of retinal carotenoids in female choice. However, supplementation differences in plasma carotenoid levels persisted and high-carotenoid birds retained significantly higher levels during the mate choice test (April, Tukey's post hoc test, *p *< 0.001, Figure 1a) that were similar to the mean levels observed among wild females (*t *= 1.49, df = 32.1, *p *= 0.15).

The effects of dietary carotenoid supplementation on the retinal carotenoid levels of these birds are presented as part of a separate study [32], but, to summarize these results, birds receiving the high-carotenoid diet had significantly higher retinal carotenoid levels (specifically of astaxanthin, galloxanthin, zeaxanthin and ε-carotene) than those fed the low-carotenoid diet.

There was no significant effect of carotenoid supplementation on body mass (time × diet: *F*_1,25 _= 0.14, *p *= 0.71, Figure 1b); however body mass changed significantly over time (*F*_3,75 _= 14.92, *p *< 0.0001). Body mass declined following capture (November vs. January, Tukey's post hoc *p *< 0.001) for both groups, then remained stable for the rest of the study (January - April, Tukey's post hoc *p *> 0.845).

Female activity levels during the mate choice trials differed significantly between the diet treatments (Wilk's λ = 0.29, *F*_1,24 _= 5.72, *p *= 0.0042), and this difference was driven primarily by a significant increase in movement of the high-carotenoid diet females (*F*_1,24 _= 14.92, *p *= 0.034). High-carotenoid females spent a mean ± S.E. 19.43 ± 1.82 min. flying and/or climbing during the trials, compared to the low-carotenoid females that spent 14.11 ± 2.09 min. moving. The treatment groups did not differ significantly in the amount of time devoted to preening (*F*_1,24 _= 0.76, *p *= 0.39) or sitting (*F*_1,24 _= 1.024, *p *= 0.32).

### Male coloration measures as a predictor of female choice

Although the manipulated coloration of the stimulus males can easily be categorized with the human visual system, these categories are unlikely to reflect how female house finches perceive male coloration [46,47]. To address this limitation, we used noise-limited receptor [48] and cone-capture [49] models of the avian visual system to calculate the contrast values and tetrahedral color-space location of each male's manipulated plumage. This analysis revealed that, despite a qualitative match to human visual perception of wild birds and hue values within the natural range of variation, our manipulated males fell outside this range when coloration was quantified using the avian visual model (additional file 1). Specifically, the red/orange, orange/yellow, and yellow experimental males tended to produce higher quantum catches for the medium-wavelength-sensitive cone than was typical of our sample of wild males (additional file 1 - Fig. S2).

Despite these limitations coloration was a significant predictor of female association time with the males. With the exception of the color space-component vector component r, all of our metrics of male coloration were significant predictors of female association time (Table 1). Although the red/orange and orange/yellow males had very similar levels of chromatic contrast when compared to red males (Table 1), their color did differ significantly when compared against one another (*ΔS *= 3.94 ± 0.34 jnds, *t *= 8.93, df = 8, *p *< 0.001). The best predictors of female choice were the tristimulus hue value and the color-space vector component θ, both of which are also significant predictors of feather carotenoid content [50]. Because of its link to avian visual perception, we chose to focus subsequent analyses on the color-space vector component θ.

**Table 1:** Male color metrics and their relationship with female association time

	Male color							
**Measure**	**red**	**red/orange**	**orange/yellow**	**yellow**	**β**	***t***	***p***	***r*^2^**

r	0.12 ± 0.01	0.12 ± 0.007	0.10 ± 0.006	0.11 ± 0.006	3.81	1.54	0.12	0.191

φ	1.28 ± 0.08	-1.39 ± 0.06	-0.616 ± 0.73	-1.01 ± 0.15	0.099	2.60	0.0098	0.216

θ	-0.48 ± 0.02	0.02 ± 0.06	0.15 ± 0.04	0.49 ± 0.10	-0.59	-4.60	< 0.0001	0.236

chromatic contrast (jnds)	-	11.0 ± 1.5	11.49 ± 0.9	15.6 ± 1.4	-0.032	-4.20	< 0.0001	0.233

achromatic contrast (jnds)	-	3.1 ± 0.7	3.6 ± 0.73	6.5 ± 1.1	-0.055	-3.21	0.0015	0.233

Hue (nm)	575.9 ± 6.7	553.2 ± 1.8	548.8 ± 1.0	524.3 ± 6.4	0.011	4.50	< 0.0001	0.244

Mean ± SE values for color measures acquired from stimulus male and the results of a linear regressions of female association time with each measure of experimental male plumage color. Chromatic and achromatic contrast values were calculated in comparison to the reddest male in each mate choice trial.

### Carotenoid status and female choice behavior

**Figure 2:**
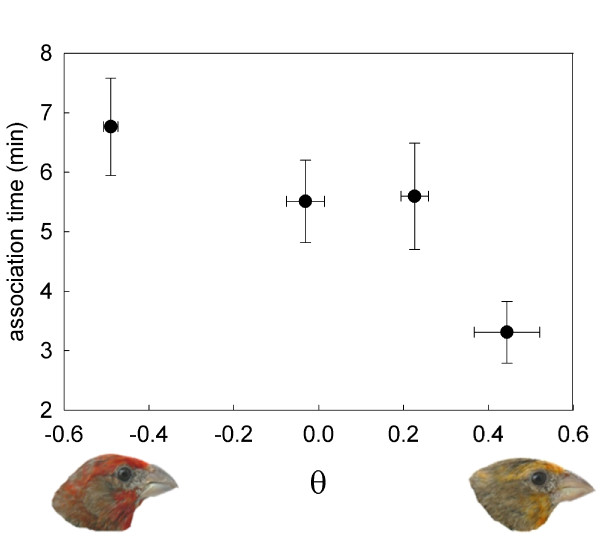
**Female association times with the color-manipulated males**. Mean ± s.e. association time of females with stimulus males of varying color. For presentation the males are grouped into four color categories, with the mean θ value of each category presented on the x-axis. Lower θ values indicate redder males.

**Figure 3:**
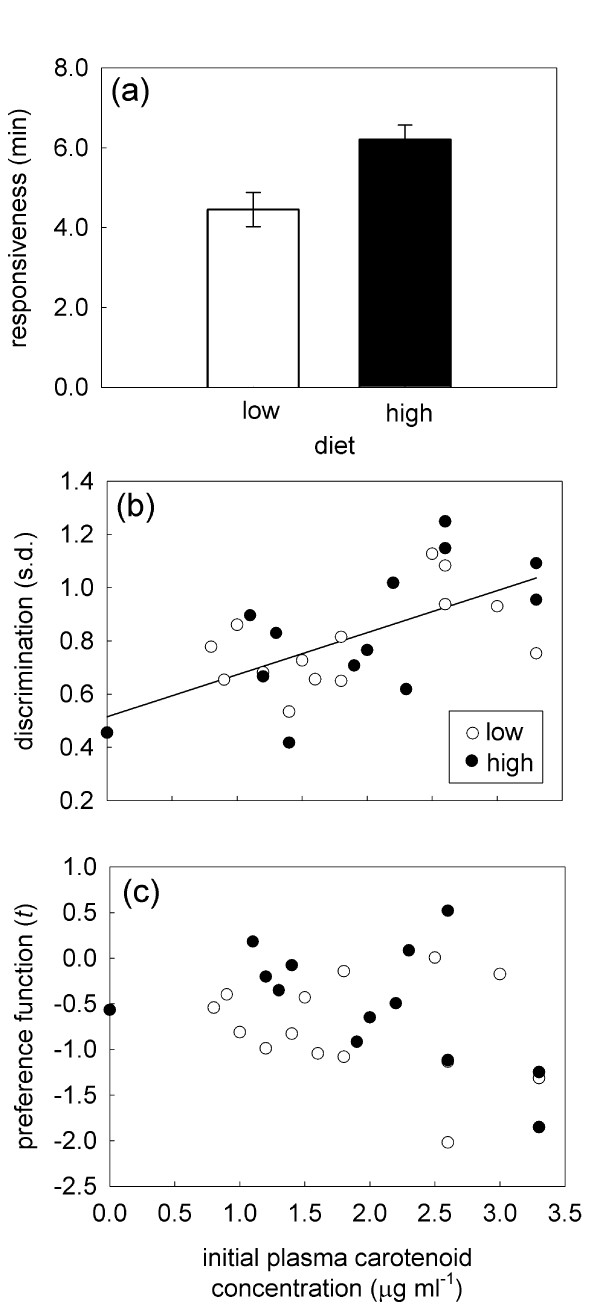
**The components of female choice in relation to diet and plasma carotenoid levels**. (a) Mean ± s.e. mate responsiveness of high- and low-carotenoid females. (b) Scatterplot illustrating the relationship between female mate discrimination and plasma carotenoid levels prior to carotenoid supplementation. (c) Scatterplot showing the female preference functions and plasma carotenoid levels prior to carotenoid supplementation. Open symbols represent the low-carotenoid females and closed symbols represent the high-carotenoid females.

Females spent significantly more time in association with the reddest males (Figure 2, *F*_1,241 _= 15.71, *p *= 0.0001); however the preference for red males did not differ significantly between females fed the low- vs. high-carotenoid diets (θ × diet: *F*_1,241 _= 0.95, *p *= 0.33). Females fed the high-carotenoid diet were significantly more responsive to males overall than were the low-carotenoid-diet females (Table 2, Figure 3a). This pattern mirrors the differences in activity level between the diet treatments, but it is not a simply a by-product of changes in activity. When activity (proportion of time moving) is included as a factor in our analysis of mate choice, diet treatments remained a significant predictor of mate responsiveness (*F*_1,23 _= 12.71, *p *= 0.0016). Mate preference functions and discrimination did not differ between the diet treatments (Table 2). However, pre-supplementation plasma carotenoid levels were a significant predictor of mate discrimination (Table 2), such that females who circulated relatively higher levels of carotenoids were more discriminating and tended bias their association time towards one or a subset of the males (Figure 3b). Female responsiveness and the mate preference function were not significantly associated with pre-supplementation plasma carotenoid levels (Table 2, Figure 3c). Time of day was a significant predictor of female association time, with females spending more time with males earlier in the day (*F*_1,241 _= 6.02, *p *= 0.018). However, there was no significant effect of time of day on color preference (θ × Time: *F*_1,241 _= 0.57, *p *= 0.45).

**Table 2:** ANCOVA analyses of the effect of diet manipulation and plasma carotenoid levels on female choice components

	Diet			Pre-supplementation plasma carotenoid levels		
**Mate choice component**	***F***	**df**	***p***	***F***	**df**	***p***

Responsiveness	9.37	1,24	**0.0054**	0.081	1,24	0.78

Discrimination	0.24	1,24	0.62	15.71	1,24	**0.00058**

Preference function	1.44	1,24	0.24	3.44	1,24	0.076

Results of univariate ANCOVAs testing the effects of dietary carotenoid supplementation and pre-supplementation plasma carotenoid levels on the three components of female choice for male plumage coloration.

Within each treatment group, total retinal carotenoid levels were not a significant predictor of mate choice behavior (High: Wilk's λ = 0.79, *F*_3,9 _= 0.78, *p *= 0.53, Low: Wilk's λ = 0.52, *F*_3,10 _= 3.02, *p *= 0.080). The repeatability of the preference for male plumage coloration did not differ significantly between diet treatments (*F*_1,25 _= 1.52, *p *= 0.23) or with retinal carotenoid accumulation within each treatment (High: *F*_1,11 _= 0.025, *p *= 0.88, Low: *F*_1,12 _= 0.60, *p *= 0.45).

We found no evidence that males behaved differently toward females from different diet treatment groups. Males were much less likely than females to terminate male-female associations during the trials (*t = *7.093, df = 27.94, *p *< 0.0001), and the frequency of male terminations did not differ significantly between the female diet treatments (*t = *-1.15, df = 22.44, *p *= 0.26). Males terminated association a mean of 5.19 ± 0.72 times per trial, while females ended associations 32.15 ± 3.73 times/trial.

## Discussion

Here we provide evidence that carotenoids, a dietary component essential for the expression of sexually selected male coloration, also influence female mate choice behavior. Specifically, female house finches receiving a carotenoid-limited diet had significantly reduced levels of mating responsiveness, and the degree of female discrimination among males was positively correlated with circulating plasma carotenoid levels at the beginning of our study, when all females were receiving a carotenoid-limited diet. In contrast, the directional preference for red male plumage coloration was unaffected by supplementation and was not significantly linked to circulating carotenoid levels at any time during the study. Below, we consider these carotenoid-dependent changes in specific components of mate choice behavior in the context of 1) physiological costs of and constraints on mate choice behavior, and 2) the adaptive value of the various mate choice behaviors [16].

### Experimental design considerations

We initially designed our study to examine the specific role of retinal carotenoid accumulation in female discrimination of colourful mates, but revealed a much more general role of carotenoids in female choice behavior. Yet, because of this specific design, it is important to consider the details of our diet and color manipulations when interpreting these more general patterns. Our dietary carotenoid manipulation consisted of a period of supplementation followed by a return to the base diet, which was intended to produce persistent changes in retinal carotenoid levels while minimizing difference in circulating carotenoid levels during the mate choice tests. However, at the time of the mate choice trials, the plasma carotenoid levels of the supplemented females were similar to the mean of wild females at the same time of year and levels in the unsupplemented females were consistent with the very lowest levels observed in the wild. Thus, the low- and high- carotenoid-diet females provide a comparison of relatively carotenoid-depleted females to individuals circulating an average level of plasma carotenoids.

We manipulated male plumage coloration following established methods [51] that provided a match to the range of wild finch coloration in simple measures of hue and qualitative human assessments. However, when we examined this coloration with a model incorporating the assumed visual capabilities of the house finch, we found that our some of experimental males (orange and yellow) fell outside of the natural range of color variation. Therefore, the consistent directional preference of females for red males in our study, although consistent with patterns of sexual selection in a number of house finch populations [36], could be the result of discrimination against orange and yellow males because they are outside the natural range of variation. Despite this limitation, our color manipulation is unlikely to have generated the diet differences in mate responsiveness and the correlation between circulating carotenoid levels and mate discrimination that we report.

### Carotenoids and mate choice constraints

In many species, mate choice is a complex and costly process requiring the location, assessment, and comparison of potential mates [16,52,53]. Moving among and interacting with potential mates depletes energetic resources [54,55], and this activity is likely to generate oxidative stress [56]. Carotenoids may facilitate active choice by countering the oxidative stress resulting from physical activity. For example, carotenoid supplementation improves the escape flight performance of zebra finches (*Taeniopygia guttata*, [57]), and antioxidant supplementation, including carotenoids, alleviates flight-induced lipid peroxidation and DNA damage in budgerigars (*Melopsittacus undulatus*; [58]). The behavioral effects of carotenoids extend beyond locomotion, and recently van Hout et al. [59] found that carotenoid supplementation of male starlings (*Sturnus vulgaris*) enhanced song production. Specifically, nest-oriented song production was increased, suggesting a link between carotenoids and reproductive behaviors in particular [59]. Consistent with this direct physiological role of carotenoids, we observed that carotenoid supplementation increased both female responsiveness to males and their general activity in the mate choice context, thus supplemented females may have been better able to meet the costs of active mate assessment and selection.

The performance of the sensory system imposes limitations on a female's ability to assess and discriminate among males. We previously found that carotenoid accumulation in the house finch retina is a significant predictor of visual discrimination in a foraging context [32], and we hypothesized that carotenoid availability may influence female choice for colorful males through carotenoid-mediated changes in color discrimination [44]. However, we found no support for this idea in the current study. Although carotenoid supplementation increased carotenoid levels in the retina, as well as significantly influenced mate choice behavior, when we looked within diet treatment groups we found no relationship between retinal carotenoid concentration and any component of mate choice behavior. Although a direct experimental manipulation of retinal carotenoids, without more general physiological effects of supplementation, would be a stronger test of this hypothesis, our data suggest it is unlikely that a retinal-carotenoid-specific mechanism is driving the changes in mate choice behavior between treatment groups. This is not altogether surprising, because the color differences between stimulus males were relatively large, easily discriminated by human observers, and likely outstripped any of the carotenoid-related shifts in discriminability. A much finer-scale manipulation that more accurately mimics natural male coloration is now needed to test for more subtle effects of retinal carotenoids on visually mediated mate selection.

### Carotenoid status and the adaptive value of mate choice

The cost and benefits of mate choice are dependent upon the context in which reproduction occurs, and females may adaptively shift their choices to balance these costs and benefits (e.g. [37,60]). Carotenoid availability and accumulation can change the context of mate choice because they can significantly influence a female's reproductive potential. Through their antioxidant and immunomodulatory effects (reviewed [61]), carotenoids can enhance female health and condition and directly promote fecundity and offspring quality [17-20,62]. In fact, a positive carotenoid balance over the course of a breeding season is a significant predictor of reproductive success in barn swallows (*Hirundo rustica*; [63]). Therefore, the reduced responsiveness of the low-carotenoid-diet females, and the correlation between discrimination and circulating carotenoid levels under dietary carotenoid limitation, may be linked to the immediate potential of these females to produce many high-quality offspring.

Grether [33,34] proposed that the value of male carotenoid-based colors as quality indicators is negatively related to the environmental availability of carotenoids. Thus, carotenoid-limited females should show stronger preferences for male carotenoid coloration than carotenoid-replete females [34]. However, we found that color preferences of female house finches were not affected by dietary carotenoid supplementation or correlated with the carotenoid accumulation ability of females. This result contradicts the predictions of Grether [33,34]; however our carotenoid manipulation occurred only during the adult stage and lacked the concomitant limitation of food availability during development that revealed these patterns in guppies [34], and our color manipulation was not exactly matched to natural male coloration. However, a stable mate preference function is not surprising because preference is generally considered innate and/or developmentally determined component of mate choice [40], which tends to vary less among individuals [41], and therefore may be less sensitive to a female's current condition [64] than other components of choice (i.e. responsiveness and discrimination). In previous work, Hill [36] found that female house finches from distinct populations, with very different patterns of male coloration, all show a common preference for red males, suggesting that red preference is an ancestral trait in the house finch that is maintained despite very different social and environmental conditions.

### Implications for the evolution of carotenoid-based coloration

We hypothesized that traits mediating carotenoid acquisition and accumulation could promote the coevolution of male ornaments and female preferences by facilitating both the production of sexually selected male coloration and female choice for those ornaments. This coevolutionary process is typically envisioned as a linkage between male ornament expression and female directional preference for that ornament [2,3,5,65]. However, we found no significant relationship between carotenoid acquisition and accumulation and the directional preferences of female house finches for male plumage coloration. Yet, the carotenoid-dependence of female responsiveness and discrimination has the potential to influence the intensity and direction of sexual selection on male carotenoid-based coloration.

Carotenoid supplemented females spent more time associating with males, and we found a significant positive correlation between pre-supplementation circulating carotenoid levels and female mate discrimination. Females that circulated relatively high plasma carotenoid levels, at the beginning of the study, later tended to focus their effort on a single male and spent less time assessing other potential mates. If we extend these observations to a natural context, we predict that females with a carotenoid-rich diet and/or the ability to accumulate high levels from dietary sources would more rapidly select a mate and thus pair up earlier in the season. Early paring female house finches tend to pair with higher-quality, redder males and enjoy higher reproductive success than later-paring birds [66-68]. This could drive a pattern of assortative mating between responsive, high-carotenoid-status females and colourful, high-carotenoid-status males, which together produce a relatively large number of offspring. Following this scenario, there should be significant selection on the traits that mediate carotenoid acquisition and accumulation in both males and females that will promote the evolution of elaborate carotenoid-based coloration. This pattern is complementary to Biard et al.'s [69] suggestion that the maternal allocation of carotenoids to egg yolks contribute to the evolution of carotenoid-based plumage coloration by facilitating the development of physiological mechanisms of carotenoid accumulation. Therefore, carotenoid-replete females may not only be more likely to pair with attractive high-carotenoid-status males, but they may also be better able to allocate carotenoids to eggs and produce high-quality attractive offspring, which may contribute similar selective pressures on the mechanisms of carotenoid acquisition and accumulation.

## Conclusions

We found that the acquisition and accumulation of carotenoids, which are nutrients important for health, female reproduction, and the production of sexually selected male coloration, are positively related to female mate responsiveness and discrimination. We suggest that the behavioral and physiological traits mediating carotenoid acquisition and accumulation provide a mechanism that may promote the coevolution of carotenoid-based sexual ornaments and female choice for those traits. The common and complementary benefits of carotenoid accumulation in males and in females may underlie the ubiquity and elaboration of carotenoid-based sexual signals in animals.

## Methods

### Experimental animals

At the beginning of their first molt into nuptial plumage (July 2009), we captured 13 hatch-year male house finches to serve as stimulus birds for our mate-choice experiment, which is a number that is consistent with previous studies of mate choice in house finches (n = 12; [36]). We trapped the males on the campus of Arizona State University in Tempe, Arizona, USA (details available in [44]) and housed them in groups of 2 in wire cages (0.6 × 0.4 × 0.3 m), in a greenhouse room that provided a natural photoperiod and semi-natural spectrum of light (i.e. the greenhouse glass blocked ultraviolet light). We fed the birds a standard maintenance diet (ZuPreem small bird maintenance diet, Premium Nutritional Products Inc. Mission, KS, USA) and tap water with a vitamin supplement (Vita-Sol, United Pet Group EIO, Tampa, FL) *ad libitum*. Because this diet contained low levels of a limited diversity of carotenoids (lutein: 1.15 ± 0.12 μg g^-1 ^and zeaxanthin: 0.52 ± 0.06 μg g^-1^), all of the males molted into uniformly drab yellow plumage that we subsequently manipulated for the mate choice trials (see below).

In November 2009, we captured 27 female house finches and housed them singly in the same greenhouse as the males, in a separate room where they were visually and acoustically isolated from the males. The females were initially maintained on a low-carotenoid sunflower seed diet for two months and then supplemented with carotenoids for our experimental treatments (see below). This study was carried out under United States Fish and Wildlife Service permit #MB088806-1 and Arizona State Game and Fish scientific collecting permit SP727468. All procedures were approved by the Institutional Animal Care and Use Committee at Arizona State University (protocol #09-1054R).

### Female dietary carotenoid manipulation and carotenoid measurement

To limit the influence of previous dietary history and storage on carotenoid availability, we maintained all of the female finches on a very low carotenoid diet of sunflower seeds (0.078 ± 0.031 μg g^-1^, lutein:zeaxanthin, 3.2:1) for the first two months after capture. This diet results in the > 95% depletion of both circulating plasma carotenoids and liver carotenoid stores [30]. In January 2010, we randomly selected 13 females and began supplementing their drinking water with carotenoids (zeaxanthin: 17.5 μg ml^-1^, OptiSharp™, DSM Inc. Heerlen, Netherlands), while the remaining 14 birds continued on the low-carotenoid diet. The supplement was given on five days per week (Monday - Friday) up until two weeks prior to the beginning of mate choice trials, at which point all birds were returned to the low-carotenoid diet. This depletion period was included in an effort to isolate the effects of increased retinal carotenoid accumulation that persists through short-term depletion from the influence of circulating carotenoids that decline rapidly [30]. However, contrary to our previous studies [30], the effects of the supplementation on circulating carotenoid levels persisted through the depletion period (see results).

To determine the effect of our diet manipulation on circulating carotenoid levels, we collected plasma samples at three time-points: 1) in January, after birds spent eight weeks on the low carotenoid diet and prior to carotenoid supplementation, 2) in March, after eight weeks of carotenoid supplementation, and 3) at the conclusion of the mate choice trials in April 2010. To measure carotenoid levels in the retina, we euthanized all females at the conclusion of the study and collected the left retina from each bird. We measured carotenoid levels in plasma and retinal tissue using high performance liquid chromatography (HPLC) following previously established protocols [44,70]. Plasma carotenoid levels are reported as μg ml^-1^, and retinal carotenoid levels are reported per whole retina (μg retina^-1^).

### Stimulus male color manipulation and measurement

To assess the influences of carotenoid accumulation and supplementation on female choice for male plumage coloration, we presented females with sets of four stimulus male finches that had their plumage coloration experimentally manipulated to vary from drab yellow to brilliant red. Following McGraw and Hill [51], we used Prismacolor^® ^art markers (Newell Rubbermaid Office Products, Oak Brook, IL, USA) to color the plumage of each male using one of four colors: red (carmine red PM-4), orange/red (yellowed orange PM-15 with carmine red PM-4), orange/yellow (yellowed orange PM-15 with canary yellow PM-19), and yellow (canary yellow PM-19). Because this coloration faded over the course of the mate choice trials, we reapplied our color treatments every two weeks.

To quantify the plumage coloration of the color manipulated males we measured the spectral reflectance of the feathers from 300 to 700 nm with an Ocean Optics (Dunedin, FL, USA) USB2000 spectrophotometer and a PX-2 pulsed xenon light source. We collected a total of nine spectra, three each from the crown, breast, and rump, and then calculated average spectra for each bird. Because the male plumage color faded, we recolored the birds biweekly, measured the fresh and faded coloration, and calculated average spectra for the intervening period. We then calculated three different sets of color metrics based upon avian visual sensitivities and traditional tristimulus scores. We used the noise-limited receptor model [48,71] and the visual system parameters from the canary (*Serinus canaria*; [72]) to calculate the chromatic and achromatic contrast of the ornamental coloration of each male in a given mate choice trial relative to the reddest male in that trial, who was given a contrast of zero (additional file 2 - supplementary methods). We calculated the tetrahedral color space location of the manipulated plumage color of each male following the methods of Stoddard et al. ([49]; additional file 2). This method defines a color as a vector in spherical coordinates, where the radius corresponds to saturation of the color, φ indicates the relative stimulation of the ultraviolet sensitive cone, and the relative stimulation of the long- and medium-wavelength sensitive cones is indicated by the θ value. For comparison, we also calculated a traditional tristimulus hue value for each male following Andersson et al. [73] and the color metrics for a sample of 75 wild male finches that we measured previously (additional file 1 - Table S1).

To examine how well each color metric predicted female choice, we used a linear-mixed model analysis, with the natural log of female association time as the dependent variable, trial nested within female id as a random effect, and compared the *R*^2 ^values of separate models with each of the color-metrics as covariates. We found that θ was the best avian visual system predictor of female preference (see results) and used this measure of coloration in all subsequent analyses.

### Choice trials and measures of choice

We quantified each female's response to the color-manipulated males in repeated (3×) mate choice trials with different sets of males but the same combination of plumage colors. The trials were conducted in a custom-built aviary (for details see [74]) that is partitioned into four visually separated flight cages that housed the stimulus males, while the female moved freely in a larger adjoining cage that gave her free visual and auditory access to the males. The female cage also contained a partition that created a "no choice" zone, where the female was out of visual contact with the males. Food (sunflower seeds) and water was available *ad libitum *at the back of the male cages and in the female "no choice" area. Each mate-choice trial lasted one hour and all trials were carried out between 0800 and 1300 hrs, from 15 March - 16 April 2010. Each female was tested only once each day, and all three trials were completed within an average of five days and a maximum of eight. Approximately 10 minutes prior to the beginning of each trial, one male of each color (red, orange/red, orange/yellow and yellow) was placed within the separate partitions of the aviary. The identity and location of the males within the aviary was randomized, such that females viewed unfamiliar males in each trial and the combinations of stimulus males differed between each female. Females were also taken from their housing cages 10 min before the trials in which they participated, and at the start of the trials were immediately released into the mate choice aviary and video recorded for one hour via four cameras, each focused on one male cage. After one hour, the female and male finches were returned to their housing cages and the next trial with different males and females was setup.

We reviewed the video recordings of each trial using the program Cowlog 1.0 [75] and quantified the amount of time, to the nearest second, that the female associated with each of the four stimulus males. We considered the female to be associating with a male when she was < 0.75 m away from him, a distance similar to previous studies in this and other finch species [42,76]. When an association ended, we recorded whether the male or female moved away. We also recorded the amount of time that females engaged in flying, sitting, preening, or were out of view in the "no choice" area. From these observations, we calculated three components of mate choice behavior: 1) responsiveness, 2) preference function, and 3) discrimination. We calculated responsiveness as the mean association time of each female across all males and trials. We calculated the preference function as the *t*-value of the regression of the natural log of association time against male coloration (θ value) following Brooks & Endler [41] and Forstmeier & Birkhead [43]. To calculate the *t*-values, we used R 2.10 [77] and the nlme package [78] to calculate the linear model lme(association time~θ*female ID, random = ~1|trial). The random factor of trial number is included to account for the non-independence of female association times within the repeated trials. We measured discrimination as the standard deviation of the mean association time for each female across all males and trials [41].

### Statistical analyses

All statistical analyses were carried out with R 2.10 [77] using the nlme package [78]. To examine the effect of dietary carotenoid supplementation on plasma carotenoid levels and body mass, we used separate repeated-measures analyses of variance (rmANOVA) with time as the within-subjects factor. To examine the effect of dietary carotenoid supplementation on retinal carotenoid concentrations, we compared levels of all six retinal carotenoid types between the dietary treatments in a multivariate analysis of variance (MANOVA). We compared average female activity in three mate choice trials (flying, sitting, and preening), per total time in view, between the diet groups in a MANOVA. Because male responsiveness to a female could significantly bias our measures of female choice, we compared the frequency (mean of the three repeated trials) that males terminated associations with high and low-carotenoid diet females with a Student's *t-*test.

We natural-log-transformed association time to meet the assumptions of parametric statistics and examined the effect of dietary carotenoid supplementation on female color preference? in a linear, mixed-effects model, with log association time as the dependent variable, diet treatment as an independent variable, male coloration (θ) and trial start time as covariates, and trial number nested within female identity as a random effect. With the exception of the diet × θ interaction, we removed all non-significant interaction terms from the final model. We then examined the relationship between the specific components of mate choice (responsiveness, preference function, and discrimination, averaged over the three mate choice trials for each female) and plasma and retinal carotenoid levels in a multivariate analysis of covariance (MANCOVA). We considered plasma carotenoid levels just prior to the diet manipulation, when all birds had been maintained on a uniform low-carotenoid diet for 2 months, to be representative of carotenoid accumulation efficiency (*sensu *[45]) and compared these levels to the components of mate choice in a MANCOVA, with diet treatment as an independent variable and pre-supplementation plasma carotenoid levels as a covariate. Because retinal carotenoid concentrations differed significantly among the diet treatments (see results), we compared total retinal carotenoid accumulation to the components of mate choice in separate MANCOVAs for each diet treatment.

We determined the repeatability of each female's choice for male coloration among the three trials following Lessells and Boag [79], by calculating separate analyses of variance for each female with association time as the dependent variable, trial number as an independent variable, and male coloration (θ) as the covariate. From these ANOVAs, we took the mean square (MS) value of θ as the within-measure error (MS_W_) and the MS of the trial term as the among-measure (MS_A_) error to calculate the repeatability. We compared repeatability between the diet treatment groups in an ANOVA and examined the Spearman rank correlations with total retinal carotenoid level within each treatment group.

## Authors' contributions

MBT and KJM conceived the project. MBT ran the experiments and analyzed the data. MBT and KJM wrote the manuscript.

## Supplementary Material

Additional file 1 **Supplementary Data**. Table S1 and figures S1 and S2 comparing the coloration of a sample of 75 wild male house finches and the experimentally manipulated stimulus males used in the mate choice trials. Click here for file

Additional file 2 **Supplementary Methods**. A detailed description of the parameters and calculations used to calculate avian visual contrasts and color-space parameters. Click here for file

## References

[B1] Andersson M Sexual Selection 1994 Princeton, NJ: Princeton University Press

[B2] Fisher RA The Genetical Theory of Natural Selection 1930 Oxford, UK: Clarendon Press

[B3] Lande R Models of Speciation by Sexual Selection on Polygenic Traits Proceedings of the National Academy of Sciences 1981 78 3721 3725 10.1073/pnas.78.6.3721 PMC319643 16593036

[B4] Grafen A Sexual selection unhandicapped by the fisher process Journal of Theoretical Biology 1990 144 473 516 10.1016/S0022-5193(05)80087-6 2402152

[B5] Kokko H Brooks R Jennions MD Morley J The evolution of mate choice and mating biases Proceedings of the Royal Society B: Biological Sciences 2003 270 653 664 10.1098/rspb.2002.2235 12769467 PMC1691281

[B6] Baldwin J Johnsen S The importance of color in mate choice of the blue crab *Callinectes sapidus* Journal of Experimental Biology 2009 212 3762 3768 10.1242/jeb.028027 19880739

[B7] Kodric-Brown A Dietary carotenoids and male mating success in the guppy: an environmental component to female choice Behavioral Ecology and Sociobiology 1989 25 393 401 401 10.1007/BF00300185

[B8] Hill GE Female House Finches Prefer Colorful Males - Sexual Selection for a Condition-Dependent Trait Animal Behaviour 1990 40 563 572 10.1016/S0003-3472(05)80537-8

[B9] Blount JD McGraw KJ Britton G, Liaaen-Jensen S, Pfander H Signal functions of carotenoid colouration Carotenoids. Volume 4: Natural Functions 2008 Switzerland: Birkhauser

[B10] Goodwin TW The Biochemistry of the Carotenoids 1984 2 New York, New York: Chapman and Hall

[B11] Chew BP Park JS Carotenoid Action on the Immune Response Journal of Nutrition 2004 134 257S 261 14704330 10.1093/jn/134.1.257S

[B12] Alonso-Alvarez C Bertrand S Devevey G Gaillard M Prost J Faivre B Sorci G An experimental test of the dose-dependent effect of carotenoids and immune activation on sexual signals and antioxidant activity American Naturalist 2004 164 651 659 10.1086/424971 15540154

[B13] Costantini D Møller AP Carotenoids are minor antioxidants for birds Functional Ecology 2008 22 367 370 10.1111/j.1365-2435.2007.01366.x

[B14] McGraw KJ Blount JD Landrum JT, Boca Raton FL Control and function of carotenoid coloration in birds: a review of case studies Carotenoids: Physical, Chemical, and Biological Functions and Properties 2009 CRC Press

[B15] McGraw KJ Nolan PM Crino OL Carotenoids bolster immunity during moult in a wild songbird with sexually selected plumage coloration Biological Journal of the Linnean Society 2011 102 560 572 10.1111/j.1095-8312.2010.01594.x

[B16] Cotton S Small J Pomiankowski A Sexual selection and condition-dependent mate preferences Current Biology 2006 16 R755 R765 10.1016/j.cub.2006.08.022 16950102

[B17] Blount JD Houston DC Møller AP Why egg yolk is yellow Trends in Ecology & Evolution 2000 15 47 49 10.1016/S0169-5347(99)01774-7 10652553

[B18] Blount JD Metcalfe NB Arnold KE Surai PF Devevey GL Monaghan P Neonatal nutrition, adult antioxidant defences and sexual attractiveness in the zebra finch Proceedings of the Royal Society B: Biological Sciences 2003 270 1691 1696 10.1098/rspb.2003.2411 12964996 PMC1691426

[B19] Biard C Surai P Møller AP Effects of carotenoid availability during laying on reproduction in the blue tit Oecologia 2005 144 32 44; 44 10.1007/s00442-005-0048-x 15868160

[B20] McGraw K Adkins-Regan E Parker R Maternally derived carotenoid pigments affect offspring survival, sex ratio, and sexual attractiveness in a colorful songbird Naturwissenschaften 2005 92 375 380 10.1007/s00114-005-0003-z 16049690

[B21] Berthouly A Helfenstein F Tanner M Richner H Sex-related effects of maternal egg investment on offspring in relation to carotenoid availability in the great tit Journal of Animal Ecology 2008 77 74 82 10.1111/j.1365-2656.2007.01309.x 18177329

[B22] Blount J Houston D Surai P Møller AP Egg-laying capacity is limited by carotenoid pigment availability in wild gulls *Larus fuscus* Proceedings of the Royal Society B: Biological Sciences 2004 271 S79 S81 10.1098/rsbl.2003.0104 15101425 PMC1809998

[B23] Goldsmith TH Collins JS Licht S The Cone Oil Droplets of Avian Retinas Vision Research 1984 24 1661 1671 10.1016/0042-6989(84)90324-9 6533991

[B24] Hart NS The visual ecology of avian photoreceptors Progress in Retinal and Eye Research 2001 20 675 703 10.1016/S1350-9462(01)00009-X 11470455

[B25] Vorobyev M Coloured oil droplets enhance colour discrimination Proceedings of the Royal Society B: Biological Sciences 2003 270 1255 1261 10.1098/rspb.2003.2381 12816638 PMC1691374

[B26] Schiedt K Bischof S Glinz E Recent Progress on Carotenoid Metabolism in Animals Pure and Applied Chemistry 1991 63 89 100 10.1351/pac199163010089

[B27] Thomson LR Toyoda Y Langner A Delori FC Garnett KM Craft N Nichols CR Cheng KM Dorey CK Elevated retinal zeaxanthin and prevention of light-induced photoreceptor cell death in quail Investigative Ophthalmology & Visual Science 2002 43 3538 12407166

[B28] Bhosale P Serban B Bernstein PS Retinal carotenoids can attenuate formation of A2E in the retinal pigment epithelium Archives of Biochemistry and Biophysics 2009 483 175 181 10.1016/j.abb.2008.09.012 18926795 PMC2683766

[B29] Knott B Berg ML Morgan ER Buchanan KL Bowmaker JK Bennett ATD Avian retinal oil droplets: dietary manipulation of colour vision? Proceedings of the Royal Society B: Biological Sciences 2010 277 953 962 10.1098/rspb.2009.1805 19939843 PMC2842729

[B30] Toomey MB McGraw KJ The effects of dietary carotenoid intake on carotenoid accumulation in the retina of a wild bird, the house finch (Carpodacus mexicanus) Archives of Biochemistry and Biophysics 2010 504 161 168 20599667 10.1016/j.abb.2010.06.033

[B31] Toomey MB Butler MW McGraw KJ Immune-system activation depletes retinal carotenoids in house finches Journal of Experimental Biology 2010 213 1709 1716 10.1242/jeb.041004 20435822

[B32] Toomey MB McGraw KJ The effects of dietary carotenoid supplementation and retinal carotenoid accumulation on vision-mediated foraging in the house finch PLoS ONE 2011 6 e21653 10.1371/journal.pone.0021653 21747917 PMC3126843

[B33] Grether GF Carotenoid limitation and mate preference evolution: a test of the indicator hypothesis in guppies (*Poecilia reticulata*) Evolution 2000 54 1712 1724 11108598 10.1111/j.0014-3820.2000.tb00715.x

[B34] Grether GF Kolluru GR Rodd FH De La Cerda J Shimazaki K Carotenoid availability affects the development of a colour-based mate preference and the sensory bias to which it is genetically linked Proceedings of the Royal Society B: Biological Sciences 2005 272 2181 10.1098/rspb.2005.3197 16191629 PMC1559943

[B35] Hill GE A Red Bird in a Brown Bag: The Function and Evolution of Colorful Plumage in The House Finch 2002 New York, NY: Oxford University Press

[B36] Hill GE Geographic variation in male ornamentation and female mate preference in the house finch: a comparative test of models of sexual selection Behavioral Ecology 1994 5 64 73 10.1093/beheco/5.1.64

[B37] Oh KP Badyaev AV Adaptive genetic complementarity in mate choice coexists with selection for elaborate sexual traits Proceedings of the Royal Society B: Biological Sciences 2006 273 1913 1919 10.1098/rspb.2006.3528 16822752 PMC1634773

[B38] Badyaev AV Hill GE Paternal care as a conditional strategy: distinct reproductive tactics associated with elaboration of plumage ornamentation in the house finch Behavioral Ecology 2002 13 591 597 10.1093/beheco/13.5.591

[B39] Jennions MD Petrie M Variation in mate choice and mating preferences: A review of causes and consequences Biological Reviews of the Cambridge Philosophical Society 1997 72 283 327 10.1017/S0006323196005014 9155244

[B40] Widemo F Sæther SA Beauty is in the eye of the beholder: causes and consequences of variation in mating preferences Trends in Ecology & Evolution 1999 14 26 31 10.1016/S0169-5347(98)01531-6 10234244

[B41] Brooks R Endler JA Female guppies agree to differ: Phenotypic and genetic variation in mate-choice behavior and the consequences for sexual selection Evolution 2001 55 1644 1655 11580024 10.1111/j.0014-3820.2001.tb00684.x

[B42] Hill GE Female house finches prefer colourful males: sexual selection for a condition-dependent trait Animal Behaviour 1990 40 563 572 10.1016/S0003-3472(05)80537-8

[B43] Forstmeier W Birkhead TR Repeatability of mate choice in the zebra finch: consistency within and between females Animal Behaviour 2004 68 1017 1028 10.1016/j.anbehav.2004.02.007

[B44] Toomey MB McGraw KJ Seasonal, sexual, and quality related variation in retinal carotenoid accumulation in the house finch (*Carpodacus mexicanus*) Functional Ecology 2009 23 321 329 10.1111/j.1365-2435.2008.01498.x

[B45] McGraw KJ Interspecific variation in dietary carotenoid assimilation in birds: Links to phylogeny and color ornamentation Comparative Biochemistry and Physiology B-Biochemistry & Molecular Biology 2005 142 245 250 10.1016/j.cbpb.2005.07.012 16129640

[B46] Bennett ATD Cuthill IC Norris KJ Sexual selection and the mismeasure of color American Naturalist 1994 144 848 860 10.1086/285711

[B47] Cuthill IC Hill GE, McGraw KJ Color Perception Bird coloration. I. Mechanisms and measurements 2006 Cambridge, MA: Harvard University Press 3 40

[B48] Vorobyev M Osorio D Bennett ATD Marshall NJ Cuthill IC Tetrachromacy, oil droplets and bird plumage colours Journal of Comparative Physiology A-Neuroethology Sensory Neural and Behavioral Physiology 1998 183 621 633 10.1007/s003590050286 9839454

[B49] Stoddard MC Prum RO Evolution of avian plumage color in a tetrahedral color space: a phylogenetic analysis of new world buntings American Naturalist 2008 171 755 776 10.1086/587526 18419340

[B50] Butler M Toomey M McGraw K How many color metrics do we need? Evaluating how different color-scoring procedures explain carotenoid pigment content in avian bare-part and plumage ornaments Behavioral Ecology and Sociobiology 2011 65 401 413 413 10.1007/s00265-010-1074-1

[B51] McGraw KJ Hill GE Carotenoid-based ornamentation and status signaling in the house finch Behavioral Ecology 2000 11 520 527 10.1093/beheco/11.5.520

[B52] Pomiankowski A The costs of choice in sexual selection Journal of Theoretical Biology 1987 128 195 218 10.1016/S0022-5193(87)80169-8 3431135

[B53] Reynolds JD Gross MR Costs and benefits of female mate choice: Is there a lek paradox? The American Naturalist 1990 136 230 243 10.1086/285093

[B54] Byers JA Wiseman PA Jones L Roffe TJ A large cost of female mate sampling in pronghorn The American naturalist 2005 166 661 8 10.1086/497401 16475083

[B55] Vitousek MN Mitchell MA Woakes AJ Niemack MD Wikelski M High costs of female choice in a lekking lizard PloS ONE 2007 2 e567 10.1371/journal.pone.0000567 17593966 PMC1891434

[B56] Powers S Deruisseau K Quindry J Hamilton K Dietary antioxidants and exercise Journal of Sports Sciences 22 81 94 (14) 10.1080/0264041031000140563 14971435

[B57] Blount JD Matheson SM Effects of carotenoid supply on escape flight responses in zebra finches, *Taeniopygia guttata* Animal Behaviour 2006 72 595 601 10.1016/j.anbehav.2005.11.014

[B58] Larcombe S Tregaskes C Coffey J Stevenson A Alexander L Arnold K The effects of short-term antioxidant supplementation on oxidative stress and flight performance in adult budgerigars *Melopsittacus undulatus* Journal of Experimental Biology 2008 211 2859 10.1242/jeb.017970 18723545

[B59] Van Hout AJ-M Eens M Pinxten R Carotenoid supplementation positively affects the expression of a non-visual sexual signal PloS one 2011 6 e16326 10.1371/journal.pone.0016326 21283591 PMC3026812

[B60] Qvarnstrom A Part T Sheldon BC Adaptive plasticity in mate preference linked to differences in reproductive effort Nature 2000 405 344 347 10.1038/35012605 10830962

[B61] McGraw K Hill GE, McGraw KJ The mechanics of carotenoid coloration in birds Bird coloration. I. Mechanisms and measurements 2006 1 Cambridge, MA: Harvard University Press Cambridge, Massachusetts 177 242

[B62] Saino N Bertacche V Ferrari RP Martinelli R Møller AP Stradi R Carotenoid concentration in barn swallow eggs is influenced by laying order, maternal infection and paternal ornamentation Proceedings of the Royal Society of London. Series B: Biological Sciences 2002 269 1729 10.1098/rspb.2002.2088 12204135 PMC1691081

[B63] Safran RJ McGraw KJ Wilkins MR Hubbard JK Marling J Positive Carotenoid Balance Correlates with Greater Reproductive Performance in a Wild Bird PLoS ONE 2010 5 e9420 10.1371/journal.pone.0009420 20195540 PMC2828481

[B64] Syriatowicz A Brooks R Sexual responsiveness is condition-dependent in female guppies, but preference functions are not BMC Ecology 2004 4 5 10.1186/1472-6785-4-5 15117410 PMC411045

[B65] Grafen A Biological signals as handicaps Journal of Theoretical Biology 1990 144 517 546 10.1016/S0022-5193(05)80088-8 2402153

[B66] Hill GE Montgomerie R Roeder C Boag P Sexual selection and cuckoldry in a monogamous songbird: implications for sexual selection theory Behavioral Ecology and Sociobiology 1994 35 193 199 10.1007/BF00167959

[B67] Hill GE Nolan PM Stoehr AM Pairing success relative to male plumage redness and pigment symmetry in the house finch: temporal and geographic constancy Behavioral Ecology 1999 10 48 53 10.1093/beheco/10.1.48

[B68] McGraw KJ Stoehr AM Nolan PM Hill GE Plumage redness predicts breeding onset and reproductive success in the House Finch: a validation of Darwin's theory Journal of Avian Biology 2001 32 90 94 10.1034/j.1600-048X.2001.320114.x

[B69] Biard C Gil D Karadas F Saino N Spottiswoode CN Surai PF Møller AP Maternal effects mediated by antioxidants and the evolution of carotenoid-based signals in birds American Naturalist 2009 174 696 708 10.1086/606021 19780651

[B70] Toomey MB McGraw KJ Modified Saponification and HPLC Methods for Analyzing Carotenoids from the Retina of Quail: Implications for Its Use as a Nonprimate Model Species Investigative Ophthalmology & Visual Science 2007 48 3976 3982 10.1167/iovs.07-0208 17724175

[B71] Siddiqi A Cronin TW Loew ER Vorobyev M Summers K Interspecific and intraspecific views of color signals in the strawberry poison frog *Dendrobates pumilio* Journal of Experimental Biology 2004 207 2471 2485 10.1242/jeb.01047 15184519

[B72] Das D Wilkie SE Hunt DM Bowmaker JK Visual pigments and oil droplets in the retina of a passerine bird, the canary *Serinus canaria*: microspectrophotometry and opsin sequences Vision Research 1999 39 2801 2815 10.1016/S0042-6989(99)00023-1 10492811

[B73] Andersson S Pryke SR Örnborg J Lawes MJ Andersson M Multiple Receivers, Multiple Ornaments, and a Trade-off between Agonistic and Epigamic Signaling in a Widowbird The American Naturalist 2002 160 683 691 10.1086/342817 18707516

[B74] Tobias MC Hill GE A test of sensory bias for long tails in the house finch Animal Behaviour 1998 56 71 78 9710463 10.1006/anbe.1998.0740

[B75] Hänninen L Pastell M CowLog: Open-source software for coding behaviors from digital video Behavior Research Methods 2009 41 472 476 10.3758/BRM.41.2.472 19363187

[B76] Burley N Krantzberg G Radman P Influence of colour-banding on the conspecific preferences of zebra finches Animal Behaviour 1982 30 444 455 10.1016/S0003-3472(82)80055-9

[B77] R Development Core Team R: A Language and Environment for Statistical Computing 2010

[B78] Pinheiro J Bates D DebRoy S Sarkar D R Development Core Team nlme: Linear and Nonlinear Mixed Effects Models 2010

[B79] Lessells CM Boag PT Unrepeatable repeatabilities: a common mistake The Auk 1987 104 116 121

