# Supplementary material for: Mate choice for a male carotenoid-based ornament is linked to female dietary carotenoid intake and accumulation
Source: BMC Evol Biol. 2012 Jan 10;12:3. doi: 10.1186/1471-2148-12-3 (PMC3315416; doi:10.1186/1471-2148-12-3)
Supplement: Additional file 1 — Supplementary Data. Table S1 and figures S1 and S2 comparing the coloration of a sample of 75 wild male house finches and the experimentally manipulated stimulus males used in the mate choice trials. [file 1471-2148-12-3-S1.PDF]

**Toomey and McGraw, Mate choice for a male carotenoid-based ornament is linked to female dietary carotenoid intake and accumulation**

# Supplementary data

Table S1. Color space location and hue of the ornamental plumage of wild male house finches (n = 75, [1]) and of the manipulated stimulus males from this study. Reflectance spectra were measured and color calculated as described in additional file 1 – supplementary methods.

| | Wild male color | | Experimental male color | | | |
|---|---|---|---|---|---|---|
| Measure | mean | range | red | red / orange | orange / yellow | yellow |
| r | 0.098±0.003 | 0.06 – 0.19 | 0.12±0.01 | 0.12±0.007 | 0.10±0.006 | 0.11±0.006 |
| φ | 1.041±0.03 | 0.06 – 1.54 | 1.28±0.08 | -1.39±0.06 | -0.616±0.73 | -1.01±0.15 |
| θ | -0.43±0.02 | -1.55 – -0.04 | -0.48±0.02 | 0.02±0.06 | 0.15±0.04 | 0.49±0.10 |
| Hue (nm) | 575.8±1.8 | 526 – 602 | 575.9±6.7 | 553.2±1.8 | 548.8±1.0 | 524.3±6.4 |

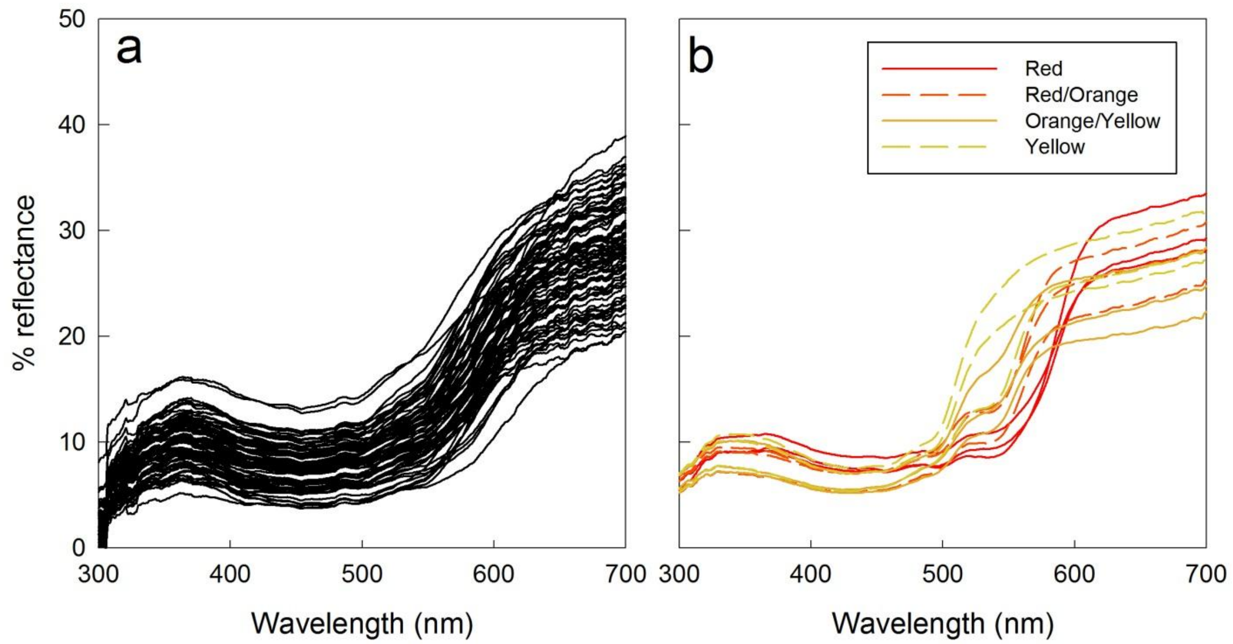


**Figure S1.** Reflectance spectra of (a) the ornamental plumage of wild male house finches (n = 75; Toomey and McGraw 2009) and (b) the color-manipulated stimulus males used in the mate choice trials. Reflectance spectra were measured as described in additional file 1 – supplementary methods.

**Toomey and McGraw, Mate choice for a male carotenoid-based ornament is linked to female dietary carotenoid intake and accumulation**

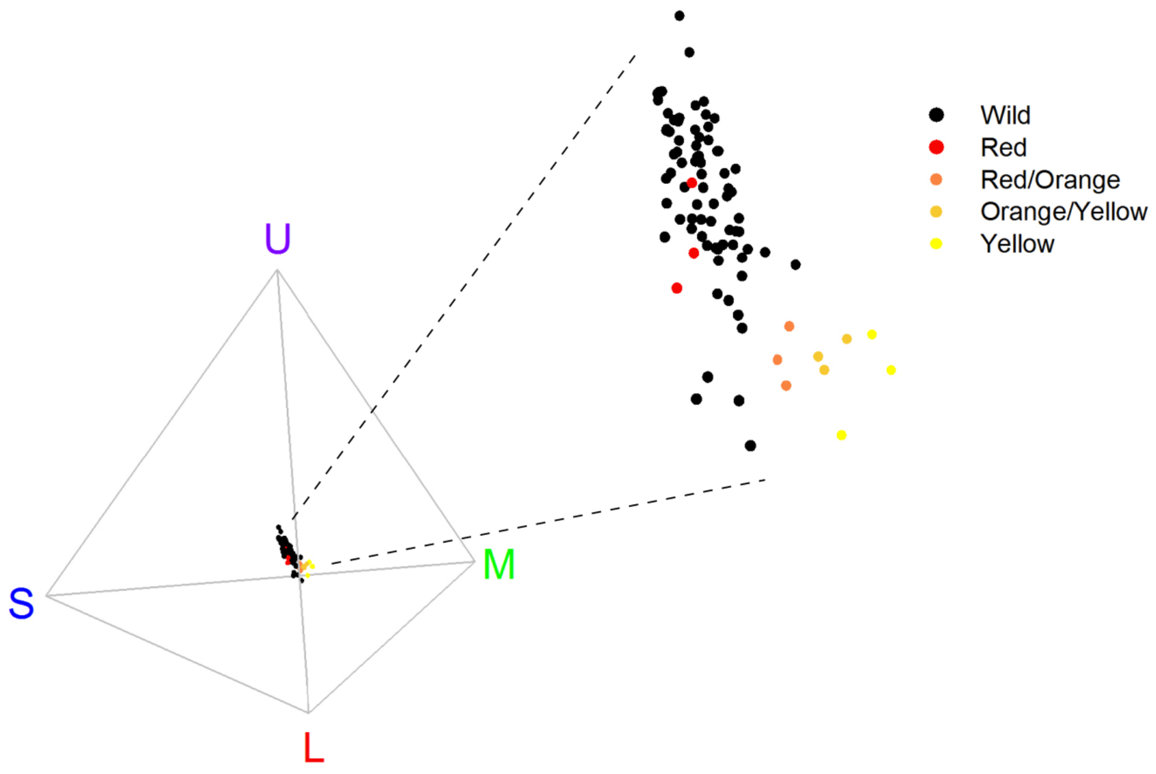


**Figure S2.** Tetrahedral color space location of the ornamental plumage of wild male house finches (black dots, n = 75, [1]) and of the color-manipulated stimulus males from this study (colored dots). Reflectance spectra were measured and color space locations calculated as described in additional file 1 – supplementary methods.


1. Toomey MB, McGraw KJ: **Seasonal, sexual, and quality related variation in retinal carotenoid accumulation in the house finch (*Carpodacus mexicanus*)**. *Functional Ecology* 2009, **23**:321-329.
