# Supplementary material for: Mate choice for a male carotenoid-based ornament is linked to female dietary carotenoid intake and accumulation
Source: BMC Evol Biol. 2012 Jan 10;12:3. doi: 10.1186/1471-2148-12-3 (PMC3315416; doi:10.1186/1471-2148-12-3)
Supplement: Additional file 2 — Supplementary Methods. A detailed description of the parameters and calculations used to calculate avian visual contrasts and color-space parameters. [file 1471-2148-12-3-S2.PDF]

**Toomey and McGraw, Mate choice for a male carotenoid-based ornament is linked to female dietary carotenoid intake and accumulation**

# Supplementary methods

### Reflectance spectra measurement

We measured reflectance spectra of the manipulated male plumage, relative to a white standard (Spectralon; Labsphere Inc., North Sutton, NH, USA), with an Ocean Optics USB2000 spectrophotometer with a PX-2 pulsed xenon light source (Ocean Optics Incorporated, Dunedin, Florida, USA). Spectra were collected from the plumage at coincident-normal geometry at a distance of 1 cm from the surface. The reflectance spectra were than binned to 1 nm intervals for subsequent analyses.

### Irradiance spectrum measurement

We measured the irradiance spectrum of light within the mate choice aviary with an Ocean Optics USB2000 spectrophotometer with a single fiber-optic probe (P400-1-UV-VIS; Ocean Optics) and a cosine-correcter with a 180° acceptance angle and a measurement surface of 6 mm in diameter (CC-3-UV; Ocean Optics). We calibrated the spectrophotometer with a standard light source (LS-1-CAL; Ocean Optics).

### Chromatic contrast calculations

To model avian visual perception of male plumage color, we followed the models proposed by Vorobyev et al. [1]. We calculated the photoreceptor quantum catch for each cone class with the following equation:

$$Q_i = \int_\lambda R_i(\lambda)S(\lambda)I(\lambda)d\lambda$$

where λ indicates wavelength, $Q_i$ is the quantum catch for the *ith* photoreceptor, $R_i(\lambda)$ is the spectral sensitivity of the *ith* photoreceptor, $S(\lambda)$ is the reflectance spectrum of the color patch, and $I(\lambda)$ is the irradiance spectrum. We calculated photoreceptor sensitivity based on physiological data from the canary as detailed below. We then calculated the log ratio of the differences in quantum catch between the reddest male and each of the other males in each trial:

$$\Delta f_i = \log\left(\frac{Q_{ia}}{Q_{back}} - \frac{Q_{ir}}{Q_{back}}\right)$$

where $Q_{ia}$ is the quantum catch of the *ith* photoreceptor in response to the reflectance spectrum of a given male, $Q_{ir}$ is the quantum catch of the ith photoreceptor in response to the reflectance spectrum of the reddest male in the trial, and $Q_{back}$ is the photoreceptor quantum catch from the background, melanin-pigmented undertail coverts.

We calculated the chromatic contrast in just-noticeable differences as follows:

$$\Delta S = \sqrt{\frac{((e_1e_2)^2(\Delta f_4 - \Delta f_3)^2 + (e_1e_3)^2(\Delta f_4 - \Delta f_2)^2 + (e_1e_4)^2(\Delta f_3 - \Delta f_2)^2 + (e_2e_3)^2(\Delta f_4 - \Delta f_1)^2 + (e_2e_4)^2(\Delta f_3 - \Delta f_1)^2 + (e_3e_4)^2(\Delta f_2 - \Delta f_1)^2)}{((e_1e_2e_3)^2 + (e_1e_2e_4)^2 + (e_1e_3e_4)^2 + (e_2e_3e_4)^2)}}$$

where $e_i$ is the Weber fraction for the given photoreceptor calculated as follows:

$$e_i = \frac{v_i}{\sqrt{n_i}}$$

where $v_i$ is the noise in a single photoreceptor, which we set to 0.05 in the model, and $n_i$ is the number of receptors of type *i*, which are given in Table S1.

## Achromatic contrast calculations

We modeled avian perception of the achromatic contrast of the carotenoid-pigmented patches following Siddiqi et al. [2], as presented by Loyau et al. [3]. We calculated the quantum catch of the double cone photoreceptors as follows:

$$Q_{dc} = \int_{\lambda} R(\lambda)S(\lambda)I(\lambda)d\lambda$$

We then calculated the log ratio of the differences in quantum catch between the reddest male and each of the other males in each trial:

$$\Delta f_{dc} = \log(\frac{Q_{dca}}{Q_{back}} - \frac{Q_{dcr}}{Q_{back}})$$

where $Q_{dca}$ is the quantum catch of the double cone in response to a given male, $Q_{dcb}$ is the quantum catch of the double cone in response to the reddest male in the trial, and $Q_{back}$ is the double cone quantum catch from the background, melanin-pigmented undertail coverts..

We calculated the achromatic contrast as follows:

$$\Delta S = \frac{|\Delta f_{dc}|}{e}$$

where the Weber fraction *e* was set to 0.05.

### Color space parameter calculations

We calculated the location of the carotenoid-pigmented patches in avian perceptual color space following the model proposed by Stoddard and Prum [4]. We calculated the quantum catch for each of the photoreceptor types $Q_i$ and the relative stimulation against the background as above. We then calculated the relative stimulation of each photoreceptor using this formula:

$$p_i = \frac{\Delta f_i}{\sum_i^1 \Delta f_i}$$

We then plotted these relative stimulations in Cartesian space using the following formulae for the XYZ coordinates:

$$X = \frac{1 - 2s - m - u}{2}\sqrt{\frac{2}{3}}$$

$$Y = \frac{-1 + 3m + u}{2\sqrt{2}}$$

$$Z = u - \frac{1}{4}$$

where *u* is the relative stimulation of the UVS/VS cone, *s* is the relative stimulation of the SWS cone, *m* is the relative stimulation of the MWS cone, and *l* is the relative stimulation of the LWS cone. We then projected these points onto a spherical coordinate system as follows:

$$r = \sqrt{X^2 + Y^2 + Z^2}$$

$$\varphi = \arctan\frac{\sqrt{X^2 + Y^2}}{Z}$$

$$\theta = \arctan\frac{Y}{X}$$

## Modeling spectral sensitivity

We modeled the absorbance spectra of the photoreceptors based upon the $\lambda_{max}$ values reported by Hart and Vorobyev [5] given in the table below and the visual pigment template of Govardovskii et al. [6] as follows:

$$S_{vp}(\lambda) = \frac{1}{\exp[69.7(a - \lambda) + \exp[28(0.922 - \lambda)] + \exp[-14.9(1.104 - \lambda) + 0.674} + S_{\beta}(\lambda)$$

where:

$$a = 0.8795 + 0.0459 \cdot \exp\left[-\frac{(\lambda_{max} - 300)^2}{11940}\right]$$

and $S_\beta(\lambda)$ equals the absorbance of the β-band of the opsin absorbance spectrum:

$$S_\beta(\lambda) = 0.26 \cdot \exp\left\{-\left[\frac{\lambda - (189 + 0.315 \cdot \lambda_{max})}{-40.5 + 0.195 \cdot \lambda_{max}}\right]^2\right\}$$

To account for the spectral tuning of the cone oil droplets, we used the cut-off values given in table S1 and model template proposed by Hart and Vorobyev [5] as follows:

$$T(\lambda) = \exp\left[-2.93 \cdot \exp[-2.89 \cdot B_{mid}(\lambda - \lambda_{cut})]\right]$$

For all models we used the ocular media transmission ($T_{ocular}(\lambda)$) of the starling [7]. Therefore, spectral sensitivity for a given photoreceptor was defined as:

$$S(\lambda) = S_{vp}(\lambda) T(\lambda) T_{ocular}(\lambda)$$

Table S2. Visual system parameters were based upon the canary (*Serinus canaria;* [8])

| Parameter | value |
|---|---|
| Cone oil droplet - $\lambda_{cut}$ and ($B_{mid}$) | |
| P-type* | 413 (0.095) |
| C-type | 414 (0.095) |
| Y-type | 506 (0.054) |
| R-type | 578 (0.054) |
| Visual pigment ($\lambda_{max}$) | |
| UVS/VS | 363 |
| SWS | 440 |
| MWS | 501 |
| LWS | 567 |
| Cone ratios (*u,s,m,l*) | 1,1,2,2 |

*P-type oil droplet parameters were not available for the canary; therefore we used values from the blue tit [9]. Published $b_{mid}$ values are not available for the P-type oil droplet; therefore we set this to the same value as the C-type oil droplet.

## References

1. Vorobyev M, Osorio D, Bennett ATD, Marshall NJ, Cuthill IC: **Tetrachromacy, oil droplets and bird plumage colours**. *Journal of Comparative Physiology A: Neuroethology, Sensory, Neural, and Behavioral Physiology* 1998, **V183**:621-633.
2. Siddiqi A, Cronin TW, Loew ER, Vorobyev M, Summers K: **Interspecific and intraspecific views of color signals in the strawberry poison frog *Dendrobates pumilio***. *Journal of Experimental Biology* 2004, **207**:2471-2485.
3. Loyau A, Gomez D, Moureau B, Théry M, Hart NS, Jalme MS, Bennett ATD, Sorci G: **Iridescent structurally based coloration of eyespots correlates with mating success in the peacock**. *Behavioral Ecology* 2007, **18**:1123-1131.
4. Stoddard MC, Prum RO: **Evolution of avian plumage color in a tetrahedral color space: a phylogenetic analysis of new world buntings**. *American Naturalist* 2008, **171**:755-776.
5. Hart NS, Vorobyev M: **Modelling oil droplet absorption spectra and spectral sensitivities of bird cone photoreceptors**. *Journal of Comparative Physiology A-Neuroethology Sensory Neural and Behavioral Physiology* 2005, **191**:381-392.
6. Govardovskii VI, Fyhrquist N, Reuter T, Kuzmin DG, Donner K: **In search of the visual pigment template**. *Visual Neuroscience* 2000, **17**:509-528.
7. Hart NS, Partridge JC, Cuthill IC: **Visual pigments, oil droplets and cone photoreceptor distribution in the European starling (*Sturnus vulgaris*)**. *Journal of Experimental Biology* 1998, **201**:1433-1446.
8. Das D, Wilkie SE, Hunt DM, Bowmaker JK: **Visual pigments and oil droplets in the retina of a passerine bird, the canary *Serinus canaria*: microspectrophotometry and opsin sequences**. *Vision Research* 1999, **39**:2801-2815.
9. Hart NS, Partridge JC, Cuthill IC, Bennett ATD: **Visual pigments, oil droplets, ocular media and cone photoreceptor distribution in two species of passerine bird: the blue tit (*Parus caeruleus* L.) and the blackbird (*Turdus merula* L.)**. *Journal of Comparative Physiology A: Neuroethology, Sensory, Neural, and Behavioral Physiology* 2000, **186**:375-387.
